# How do infants enter and move through the care and family justice systems in Wales? Protocol for a population-based data linkage study.

**DOI:** 10.23889/ijpds.v7i3.1811

**Published:** 2022-08-25

**Authors:** Laura Cowley, Laura North, Karen Broadhurst, Stefanie Doebler, Linda Cusworth, Lucy Griffiths

**Affiliations:** 1 Swansea University; 2 Lancaster University

## Objectives

We aim to determine the proportion of infants entering care in Wales via the two primary legal routes (section 76 of the Social Services and Wellbeing (Wales) Act 2014, and section 31 of the Children Act 1989), and associations between mode of entry and infant characteristics and outcomes.

## Approach

This is a longitudinal cohort study using routinely collected data held in the Secure Anonymised Information Linkage (SAIL) Databank. We will link the Looked After Children dataset with family justice (Cafcass Cymru) data on section 31 proceedings, to explore pathways through the care and family justice systems for infants aged <1 year entering care in Wales between 2012 and 2019. We will follow up each child for two years from the date of their entry into care to track legal outcomes (final legal orders) and placement outcomes (placement type, number of placements, and re-entry into the care system).

## Results

Descriptive statistics will include frequencies and proportions of infants who initially enter the care system via voluntary arrangements (section 76) and care proceedings (section 31), by age, year, local authority, and category of need. We will describe the proportion and characteristics of those with voluntary arrangements who later become the subject of care proceedings, and the distribution of final legal orders and placement types by initial route of entry to care. We will use funnel plots to investigate variation between local authorities. We will use linear regression to test for statistically significant differences in the proportions of infants entering care via the two different routes over time, and chi-square tests to investigate associations between mode of entry and infant characteristics and outcomes.

## Conclusion

There is limited information on the care journeys of children in Wales at the individual level. This study will help us to understand the patterns of use of voluntary arrangements for infants over time, the proportion subsequently involved in section 31 applications and the impact on outcomes for children.

